# Supplementary material for: First Glance of Molecular Profile of Atypical Cellular Angiofibroma/Cellular Angiofibroma with Sarcomatous Transformation by Next Generation Sequencing
Source: Diagnostics (Basel). 2020 Jan 9;10(1):35. doi: 10.3390/diagnostics10010035 (PMC7169459; doi:10.3390/diagnostics10010035)
Supplement: Supplementary file 1 [file diagnostics-10-00035-s001.pdf]

Figure S1: Immunohistochemical and fluorescence in situ hybridization(FISH) result of six cases of atypical angiofibroma/angiofibroma with sarcomatous transformation and two cellular angiofibroma control cases.

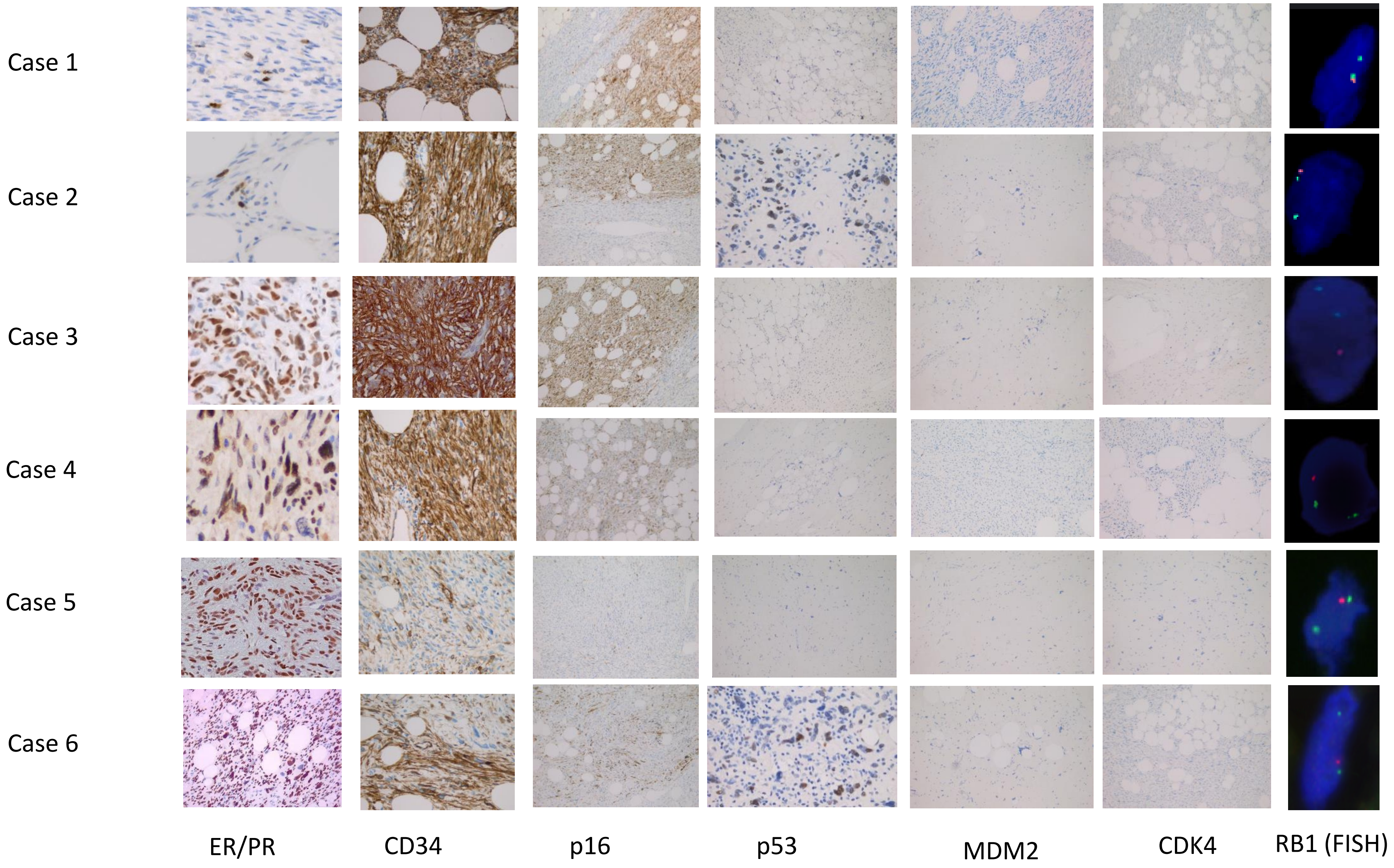

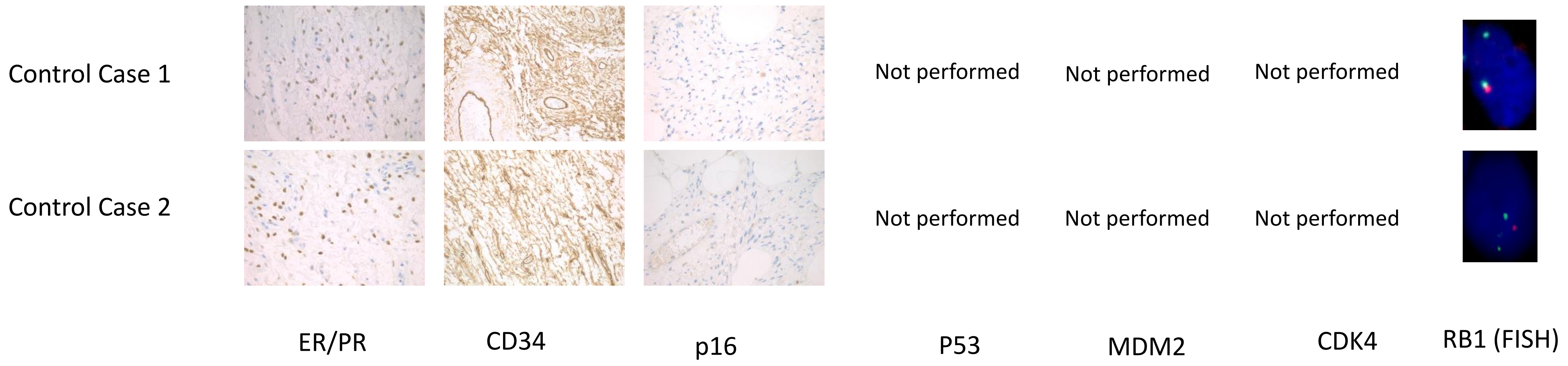
Control Case 1
Not performed
Not performed
Not performed
Control Case 2
Not performed
Not performed
Not performed
ER/PR
CD34
p16
P53
MDM2
CDK4
RB1 (FISH)
